# Association of neutrophil-to-lymphocyte ratio (NLR) with the prognosis of first attack neuromyelitis optica spectrum disorder (NMOSD): a retrospective cohort study

**DOI:** 10.1186/s12883-021-02432-0

**Published:** 2021-10-08

**Authors:** Haojie Xie, Yi Zhao, Chunyang Pan, Jinwei Zhang, Yongyan Zhou, Yanfei Li, Ranran Duan, Yaobing Yao, Zhe Gong, Junfang Teng, Yanjie Jia

**Affiliations:** Department of Neurology, the First Affiliated Hospital of Zhengzhou University, Zhengzhou, 450052 China

**Keywords:** Neuromyelitis optica spectrum disorder, Neutrophil-to-lymphocyte ratio, First attack, Prognosis, Extended disability status scale

## Abstract

**Background:**

To investigate the relationship between the neutrophil-to-lymphocyte ratio (NLR) and prognosis after the first attack of optic neuromyelitis optica spectrum disorder (NMOSD).

**Methods:**

In this retrospective study, we included the medical records of 324 patients with first episode NMOSD and collected data on clinical parameters. Follow-up extended disability status scale (EDSS) score and relapse rate were analyzed using logistic regression models to determine the independent effect of NLR on outcomes; receiver operating characteristic (ROC) curves were applied to analyze the predictive value of NLR for the prognosis of NMOSD. Interaction and stratification analyses were used to explore the association between NLR and prognosis of patients with NMOSD, and Kaplan-Meier analysis was used to investigate the relationship between NLR and outcome. The association between NLR level with relapse rate and poor recovery was assessed by a Cox regression analysis.

**Results:**

Patients in the high-NLR group had significantly higher EDSS scores and relapse rates at follow-up (both, *P* < 0.001) than did those in the low-NLR group. Univariate analysis showed revealed that NLR was significantly associated with relapse (odds ratio [OR] = 1.28, 95% confidence interval [CI]: 1.16–1.41, *P* < 0.001) and poor recovery (OR = 1.32, 95% CI: 1.20–1.46, P < 0.001), and these associations remained significant, even after multifactorial analysis (OR = 1.33, 95% CI: 1.11–1.59, *P* = 0.002; OR = 1.23, 95% CI: 1.06–1.43, *P* = 0.007, respectively). Stratified analysis showed that sex, platelet-to-lymphocyte ratio (PLR) level, and lymphocyte-to-monocyte technical ratio (LMR) level were strongly associated with relapse owing to elevated NLR; Kaplan-Meier survival curve analysis showed that the median time to relapse was significantly lower in the high-NLR group than in the low-NLR group (*P* < 0.001). A multivariate analysis showed a significant relationship between NLR level with relapse (HR = 1.07, 95%CI: 1.03–1.10, *P* = 0.001) and poor recovery (HR = 1.08, 95%CI: 1.04–1.11, P = 0.001).

**Conclusions:**

NLR may be used as a prognostic indicator for first onset NMOSD, and a high NLR may be significantly associated with high relapse rates and poor recovery.

## Background

Neuromyelitis optica spectrum disorder (NMOSD) is an autoimmune inflammatory demyelinating disease of the central nervous system (CNS) [1]. The pathogenesis of NMOSD is not fully understood, and it is currently believed that many inflammatory factors are altered during its pathogenesis, and the main pathological manifestations include central nervous system inflammation, demyelination, and astrocyte damage, with pro-inflammatory cytokines and innate immune cells playing a key role in its development [2]. Many researchers have suggested significant biomarkers for the prognosis of NMOSD patients, such as AQP4 antibody specific marker of NMOSD and MOG antibody in some patients with negative AQP4 antibody. Meanwhile, some sensitive biomarkers with differential significance are also being investigated, such as serum and cerebrospinal fluid N-acetyl aspartic acid which can differentiate multiple sclerosis and NMOSD [3]. In addition, the prognostic significance of cells with cytokines such as CD16+, CD56+, NK cells and CX3CL1 has also been pointed to [4]. However, there remain no widely unified biomarkers for predicting recurrence and prognosis. The high rate of relapse and disability associated with NMOSD, with the advent of targeted drugs, is crucial for the accurate diagnosis of patients with NMOSD. In addition, sensitive biomarkers for monitoring disease activity must be investigated to assess outcomes and prognoses.

Peripheral blood neutrophil-to-lymphocyte ratio (NLR), which is more reflective of the inflammatory state of an organism than are inflammatory indices represented by leukocytes and their subtypes, has been shown in several studies to be correlated with the prognosis of malignancies, inflammatory bowel disease, endocrine diseases, rheumatologic disease onset and progression, and the severity of acute coronary syndromes [5–11]. It has been proposed that elevated NLR is a complementary and independent marker of the severity of multiple sclerosis-related neurological dysfunction and the corresponding magnetic resonance imaging findings, respectively [12]. In relapsing-remitting multiple sclerosis, NLR correlates with disease activity [13]. However, a recent study proposed that NLR should not be used as a marker of disease activity and disability in for patients with multiple sclerosis [14]. Nevertheless, previous studies have indicated that NLR can be used to assess disease activity in patients with NMOSD because it could reflect the exacerbation of neurological disability [15], but the relationship between NLR and prognosis of for patients with NMOSD has not been discussed in previous studies; therefore, the aim of this study was to investigate the correlation between NLR and prognosis of NMOSD patients after their first attack.

## Methods

### Clinical data

**Fig. 1:**
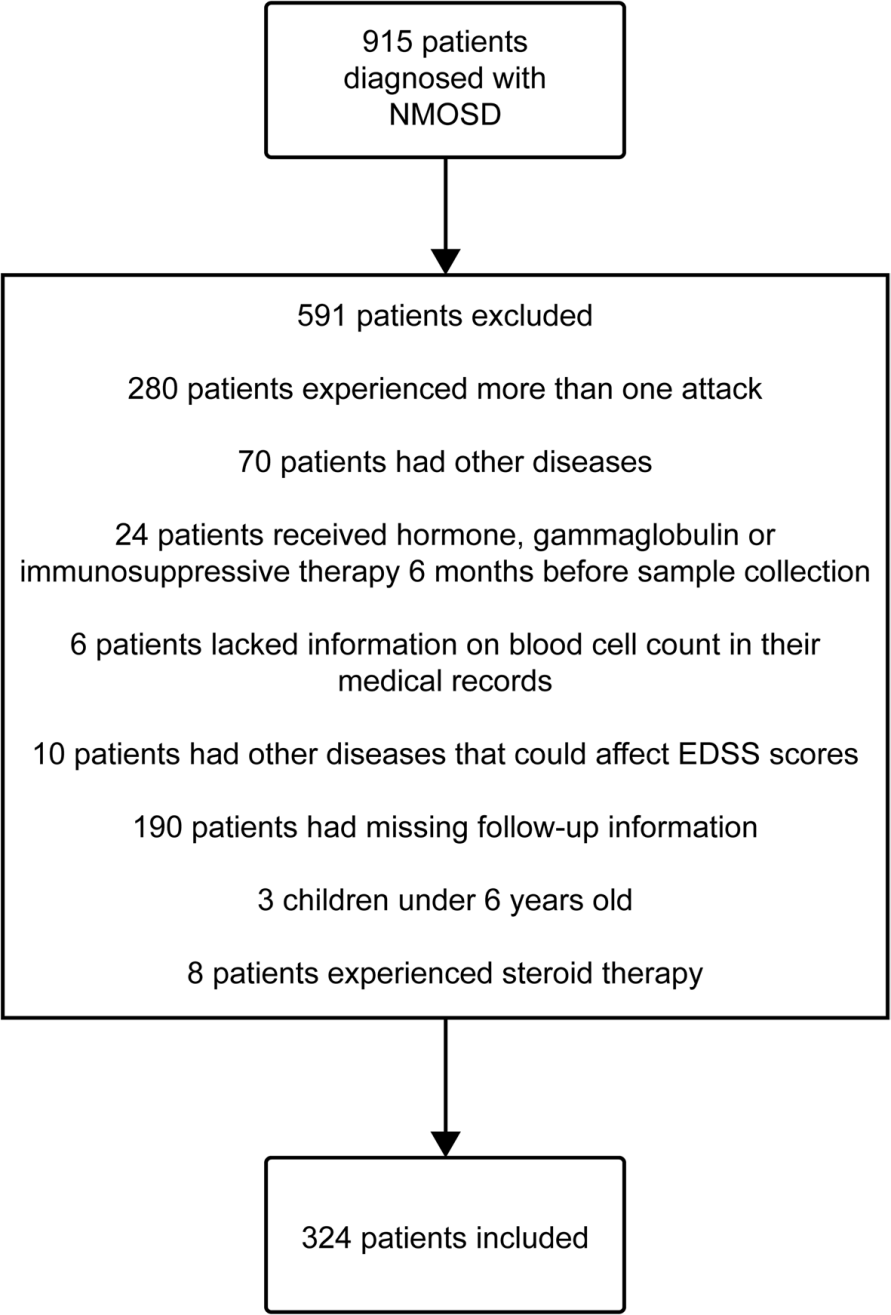
Flow chart showing the patient screening process

In this cohort study, we enrolled 912 patients from the Department of Neurology of the First Affiliated Hospital of Zhengzhou University from June 2013 to March 2020. We conducted the last follow-up on January 20, 2021, and the median follow-up period lasted was 1290 days. All patients met the NMOSD International Consensus Diagnostic Criteria [1]. All patients were selected for screening, and 324 patients were finally included in the study (Fig. 1: Flowchart of participant selection) after applying the following exclusion criteria: (1) occurrence of more than one attack of NMOSD; (2) presence of comorbid autoimmune diseases, liver disease, kidney disease, cardiovascular disease, malignancy, etc.; (3) recipience of treatment with hormones, gamma-globulins, and immunosuppressive drugs in the 6 months prior to admission; (4) lack of information on blood counts or missing follow-up information in medical records; (5) presence of other neurological diseases that may or may not affect expanded disability status scale (EDSS) scores; (6) loss of blood cells; (7) children under 6 years old; and (8) previous steroid treatment.

### Data acquisition

Baseline clinical information, including demographic characteristics (sex, age, underlying disease), medical history (past medical history, first symptoms, initial EDSS score), test data (blood counts, presence of anti-AQP4 antibodies), imaging characteristics (number of spinal cord-involved segments), and treatment information (hormones, gamma-globulins, and immunosuppressants) were obtained from the case report form at admission. Follow-up data were obtained through annual outpatient interviews or quarterly telephone interviews. Two experienced neurologists independently assessed the severity and NMOSD relapse according to EDSS scores at the initial visit and final follow-up, respectively, and obtained the initial and follow-up EDSS scores. In addition, all patients included in the study received clear follow-up data. The project was approved by the ethics committee of Zhengzhou University (ethics review number: 2019-KY-018). We confirm that all methods were performed in accordance with the relevant guidelines and regulations.

### Laboratory data

All blood samples acquired for biomarker identification were analyzed in the biochemistry laboratory of the First Affiliated Hospital of Zhengzhou University. Fasting venous blood samples of all patients were collected early in the morning, on the day after admission, for routine blood tests. Platelet count, neutrophil count, lymphocyte count, and monocyte count were recorded, and NLR, platelet-to-lymphocyte ratio (PLR), and lymphocyte-to-monocyte technical ratio (LMR) were calculated; blood counts were analyzed using an automated analyzer (Beckman Coulter Hematology Analyzer LH750, USA). Transfected live cells were used to measure the AQP4 antibodies.

### Outcomes

The degree of disability in patients with NMOSD was assessed using the EDSS score [16], which can be used to visually score mild (EDSS score 0–3.5) or moderate/severe (EDSS score 4–9.5) disability [17]. We chose an EDSS score of 4.0 as a disability cut-off, consistent with previous studies [18, 19]. Therefore, in this study, subjects with a follow-up EDSS score of > 3.5 (EDSS ≥4) were defined to show poor recovery, and the remaining subjects were defined to show good recovery. Relapse was defined according to the following criteria: appearance of new neurological symptoms or exacerbation of existing neurological symptoms lasting for > 24 h, time to the last onset of > 1 month, and imaging confirmation of a new lesion. The relapse and poor recovery mentioned in our article refer to patients with relapse and a follow-up EDSS ≥4.

### Statistical methods

Statistical analyses were performed using SPSS 26.0. The data obtained in this study were tested for normality, and the data that conformed to the normal distribution are expressed as mean ± standard deviation (x ± s); t-test for independent samples was used for comparison between two groups. Data that did not conform to normal distribution are expressed as median (interquartile range: M [Q1, Q3]), and the Mann–Whitney U test was used for comparison between the two groups. Categorical count data were expressed as the number of cases (percentage: n [%]), and the chi-square test or Fisher’s exact test was used for comparison between groups. According to the median, NLR was divided into two groups, namely the high NLR group and low NLR group. In a logistic regression analysis, relapse and poor recovery were taken as dependent variables. The covariates are as follows: age of onset, sex, NLR, PLR, LMR, LETM, AQP4 status, initial EDSS, hypertension, diabetes, therapeutic regimens (Corticosteroids, Immunoglobulins, Immunosuppressants), and disease-modifying treatments during the follow-up period. Univariate logistic regression models were used to assess whether NLR or other covariates had an independent effect on the poor recovery and relapse, and multivariate logistic regression models were used to analyze the independent effect of NLR on relapse rate and follow-up EDSS scores; we adjusted multiple confounders to analyze the stability of the associations between NLR level and relapse and poor recovery. Variables, including NLR, PLR, and LMR, were considered in the basic model, because they are considered biomarkers of inflammatory activity in blood cell counts. Previous studies have suggested that sex, age, long-segment transverse myelitis, and anti-AQP4 antibody titers may affect the prognosis of patients with NMOSD [20–22]. Based on these findings, we adjusted for sex, age, long-segment transverse myelitis, and the presence of anti-AQP4 antibodies. A model with adjustment I was obtained. Finally, we examined the associations between NLR level and outcomes by further adjusting for treatment methods, and complications in the Adjusted II model. The significance level was established at *p* < 0.05. The predictive value of NLR for the prognosis of patients with NMOSD was analyzed using ROC curves, and the optimal cut-off point for continuous variables was determined based on the maximum Youden index. Next, we divided the variables into different subgroups and performed subgroup and interaction analyses. A Kaplan-Meier analysis was performed for the accumulation of NMOSD relapse occurrences. A univariate Cox proportional hazards model was used to screen variables with *P* ≤ 0.1, and a multivariate Cox regression model was used to analyze the predictive strength of NLR levels on the timing of the first relapse of NMOSD. Results were expressed as hazard ratios (HRs).

## Results

The whole NLR was non-normally distributed, with quartiles of 2.59 (1.63, 4.28). The included patients were 7 to 84 years old (mean, the average age was 43 years); old, the quartile was 44 (30,56), and 25 patients under 18 years old accounted for 7.7% of the total. The high-NLR group showed experienced a significantly higher relapse rate during the follow-up period (*P* < 0.001), higher initial and follow-up EDSS scores (P < 0.001), higher PLR (P < 0.001), and lower LMR (P < 0.001) compared to the low-NLR group. No significant differences were observed in the remaining parameters (Table 1).

**Table 1:** Demographics and clinical characteristics

	Low-NLR group (*n* = 162)	High-NLR group (n = 162)	*P* value
Age, years,mean ± SD	41.65 ± 16.58	45.15 ± 17.32	0.064
Male sex, no. (%)	39 (24.1%)	54 (33.3%)	0.065
Hypertension, no. (%)	18 (11.1%)	27 (16.7%)	0.148
Diabetes, no. (%)	9 (5.6%)	10 (6.2%)	0.813
Initial EDSS, median (IQR)	4 (3, 5)	4 (5, 7)	< 0.001
Initial symptoms (%)			
Optic neuritis, no. (%)	39 (24.1%)	35 (21.6%)	0.597
Acute myelitis, no. (%)	75 (46.3%)	75 (46.3%)	–
Optic neuritis with Acute myelitis, no. (%)	16 (9.9%)	21 (13.0%)	0.382
LETM, no. (%)			0.062
Not tested	54 (33.3%)	50 (30.9%)	
Yes	75 (46.3%)	90 (55.6%)	
No	33 (20.4%)	22 (13.5%)	
Anti-AQP4 antibody, no. (%)			0.337
Not tested	42 (25.9%)	11 (6.8%)	
Negative	44 (27.2%)	47 (29.0%)	
Positive	76 (46.9%)	104 (64.2%)	
Corticosteroids, no. (%)	155 (95.7%)	155 (95.7%)	–
Immunoglobulins, no. (%)	14 (8.6%)	19 (11.7%)	0.358
Immunosuppressants, no. (%)	52 (32.1%)	59 (36.4%)	0.245
Blood platelet count (× 10^9^/L), median (IQR)	214 (178,256)	227.5 (185.75, 284)	0.147
Neutrophil count (× 10^9^/L), median (IQR)	3.2 (2.59, 4.15)	6.01 (4.45, 8.40)	< 0.001
Lymphocyte count (× 10^9^/L), median (IQR)	2 (1.7, 2.4)	1.2 (0.86, 1.71)	< 0.001
Monocyte count (× 10^9^/L), median (IQR)	0.45 (0.36, 0.59)	0.5 (0.34, 0.65)	0.428
NLR, median (IQR)	1.63 (1.28, 2.05)	4.28 (3.01, 6.72)	< 0.001
PLR, median (IQR)	104.63(82.54, 135.62)	182.20 (122.06, 252.75)	< 0.001
LMR, median (IQR)	4.4 (3.4, 5.53)	2.77 (1.20, 4.22)	< 0.001
Follow-up EDSS, median (IQR)	2 (2, 3)	4 (3, 6)	< 0.001
Relapse, no. (%)	33(20.4%)	104(64.2%)	< 0.001
Interval, days, median (IQR)	1311 (959, 1777)	1233 (809, 1751)	0.223
DMT, no. (%)	21(13.0%)	24(14.8%)	0.63
Azathioprine	9(5.6%)	12(7.4%)	0.498
Mycophenolate mofetil	7(4.3%)	4(2.5%)	0.357
Cyclophosphamide	2(1.2%)	3(1.9%)	1
Intravenous immunoglobulin	1(0.6%)	2(1.2%)	1
Rituximab	1(0.6%)	2(1.2%)	1

*EDSS* Extended disability status scale, *LMR* Lymphocyte-to-monocyte technical ratio, *NLR* Neutrophil-to-lymphocyte ratio, *PLR* Platelet-to-lymphocyte ratio, *LETM* longitudinally extensive transverse myelitis, *AQP4* aquaporin-4, *DMT* disease-modifying treatment started during the follow-up

To investigate the potential factors influencing the prognosis of NMOSD, we performed a univariate analysis, which showed revealed that hypertension (OR = 0.39, 95% CI: 0.20–0.75, *P* = 0.004), initial EDSS score (OR = 1.13, 95% CI: 1.08–1.36, *P* = 0.001), and NLR (OR = 1.28, 95% CI: 1.16–1.41, *P* < 0.001) were significantly associated with relapse. In addition, we found that the initial EDSS score (OR = 1.22, 95% CI: 1.08–1.37, P = 0.001) and NLR (OR = 1.32, 95% CI: 1.20–1.46, P < 0.001) were significantly associated with poor patient recovery, as detailed in Table 2.

**Table 2:** Univariate analysis of factors affecting the prognosis of patients with NMOSD

Variable	Statistical values	Relapse		Poor recovery	
		OR (95% CI)	P value	OR (95% CI)	P value
Age, years	43.40 ± 17.03	1.00 (0.99, 1.01)	0.834	0.99 (1.00, 1.01)	0.27
Male sex, no. (%)	93 (28.7%)	1.42 (0.87, 2.3)	0.159	1.36 (0.83, 2.23)	0.218
Hypertension, no. (%)	45 (13.9%)	0.39 (0.20, 0.75)	0.004	0.76 (0.40, 1.45)	0.411
Diabetes, no. (%)	19 (6%)	1.63 (0.60, 4.41)	0.344	0.63 (0.25, 1.59)	0.325
Initial EDSS	5 (3, 6)	1.13 (1.08, 1.36)	0.001	1.22 (1.08, 1.37)	0.001
Anti-AQP4 antibody, no. (%)	180 (66.4%)	0.68(0.41, 1.14)	0.146	0.96 (0.57, 1.61)	0.876
LETM	165 (75%)	0.76 (0.41, 1.42)	0.388	0.93 (0.50, 1.73)	0.811
Corticosteroids, no. (%)	310 (95.7%)	1.03 (0.35, 3.02)	0.965	0.46 (0.13, 1.67)	0.236
Immunoglobulins, no. (%)	33 (10.2%)	1.00 (0.48, 2.06)	0.986	1.18 (0.55, 2.53)	0.67
Immunosuppressants, no. (%)	111 (34.3%)	0.94 (0.59, 1.50)	0.801	0.99 (0.61, 1.59)	0.955
NLR	2.59 (1.63, 4.28)	1.28 (1.16, 1.41)	< 0.001	1.32 (1.20, 1.46)	< 0.001
PLR	131 (98, 199)	1.01 (1.00, 1.02)	< 0.001	1.01 (1.01, 1.02)	< 0.001
LMR	3.68 (2.56, 5.00)	1.01 (0.97, 1.06)	0.529	1.02 (0.98, 1.06)	0.458
DMT	45(13.9%)	1.12(0.59,2.13)	0.724	0.96 (1.50,1.83)	0.955

*EDSS* Extended disability status scale, *LMR* Lymphocyte-to-monocyte technical ratio, *NLR* Neutrophil-to-lymphocyte ratio, *PLR* Platelet-to-lymphocyte ratio, *LETM* longitudinally extensive transverse myelitis, *AQP4* aquaporin-4, *DMT* disease-modifying treatment started during the follow-up

The multifactorial analysis results revealed that the higher the value of NLR, the higher the probability of relapse and poor recovery and revealed the relationship of NLR with relapse (OR = 1.33, 95% CI: 1.11–1.59, *P* = 0.002) and with poor recovery (OR = 1.23, 95% CI: 1.06–1.43, *P* = 0.007). The basic model included NLR, PLR, and LMR, and the results demonstrated the relationship of NLR with relapse (OR = 1.35, 95% CI: 1.14–1.59, *P* < 0.001) and poor recovery (OR = 1.24, 95% CI: 1.01–1.42, *P* = 0.003). The model with adjustment I showed indicated the relationship association between of NLR with and relapse (OR = 1.33, 95% CI: 1.21–1.57, P < 0.001) and poor recovery (OR = 1.23, 95% CI: 1.06–1.43, P < 0.001). Based on the adjusted model II, NLR was found to be associated with relapse (OR = 1.33, 95% CI: 1.11–1.59, *P* = 0.002) and poor recovery (OR = 1.23, 95% CI: 1.06–1.43, *P* = 0.007), as detailed in Tables 3 and 4.

**Table 3:** Adjusted logistic regression models for association of NLR with the relapse of NMOSD

Variables	Basic model		Adjust-I model		Adjust-II model	
	OR (95% CI)	*P* value	OR (95% CI)	P value	OR (95% CI)	P value
NLR	1.35(1.14, 1.59)	< 0.001	1.33 (1.21, 1.57)	< 0.001	1.33(1.11, 1.59)	0.002
PLR	1.00(0.99, 1.01)	0.213	1.00 (0.99, 1.02)	0.251	1.00 (0.99, 1.01)	0.206
LMR	0.98(0.91, 1.05)	0.586	0.98 (0.91, 1.05)	0.591	0.99 (0.92, 1.07)	0.836
Sex			0.86 (0.40, 1.86)	0.703	0.93 (0.42, 2.06)	0.863
Age			1.00 (0.98, 1.02)	0.607	1.00 (0.98, 1.02)	0.645
LETM			0.97 (0.46, 2.06)	0.945	1.00 (0.47, 2.17)	0.981
Anti-AQP4 antibodies			0.85 (0.41, 1.76)	0.667	0.84 (0.40, 1.76)	0.649
Initial EDSS			1.06 (0.9, 1.27)	0.447	1.06 (0.88, 1.28)	0.526
Hypertension					0.60 (0.22, 1.66)	0.328
Diabetes					2.09 (0.46, 9.53)	0.343
Corticosteroids					1.65 (035, 7.72)	0.528
Immunoglobulins					1.34 (0.43, 4.17)	0.611
Immunosuppressants					0.97 (0.49, 1.94)	0.932
DMT					1.44 (0.54,3.87)	0.466

*EDSS* Extended disability status scale, *LMR* Lymphocyte-to-monocyte technical ratio, *NLR* Neutrophil-to-lymphocyte ratio, *PLR* Platelet-to-lymphocyte ratio, *LETM* longitudinally extensive transverse myelitis, *AQP4* aquaporin-4, *DMT* disease-modifying treatment started during the follow-up

**Table 4:** Adjusted logistic regression models for the association of NLR with poor recovery in patients with NMOSD

Variables	Basic model		Adjust-I model		Adjust-II model	
	OR (95% CI)	P value	OR (95% CI)	P value	OR (95% CI)	P value
NLR	1.24 (1.01, 1.42)	0.003	1.23 (1.06, 1.43)	0.005	1.23 (1.06, 1.43)	0.007
PLR	1.00 (0.99, 1.01)	0.359	1.00 (0.99, 1.01)	0.416	1.00 (0.99, 1.01)	0.360
LMR	1.04 (0.95, 1.14)	0.381	1.04 (0.95, 1.15)	0.393	1.05 (0.95, 1.16)	0.370
Sex			0.82 (0.39, 1.75)	0.61	0.84 (0.39, 1.85)	0.669
Age			1.00 (0.98, 1.02)	0.914	1.00 (0.98, 1.02)	0.887
LETM			1.36 (0.65, 2.84)	0.412	1.28 (0.60, 2.70)	0.524
Anti-AQP4 antibodies			0.99 (0.49, 2.00)	0.985	0.98 (0. 47, 1.99)	0.932
Initial EDSS			1.14 (0.96, 1.35)	0.12	1.14 (0.95, 1.36)	0.167
Hypertension					0.58 (0.22, 1.52)	0.269
Diabetes					0.58 (0.13, 1.87)	0.294
Corticosteroids					1.30 (0.27, 6.12)	0.763
Immunoglobulins					1.83 (0.57, 5.90)	0.312
Immunosuppressants					0.93 (0.47, 1.83)	0.822
DMT					1.41 (0.54, 3.72)	0.487

*EDSS* Extended disability status scale, *LMR* Lymphocyte-to-monocyte technical ratio, *NLR* Neutrophil-to-lymphocyte ratio, *PLR* Platelet-to-lymphocyte ratio, *LETM* longitudinally extensive transverse myelitis, *AQP4* aquaporin-4, *DMT* disease-modifying treatment started during the follow-up

**Fig. 2:**
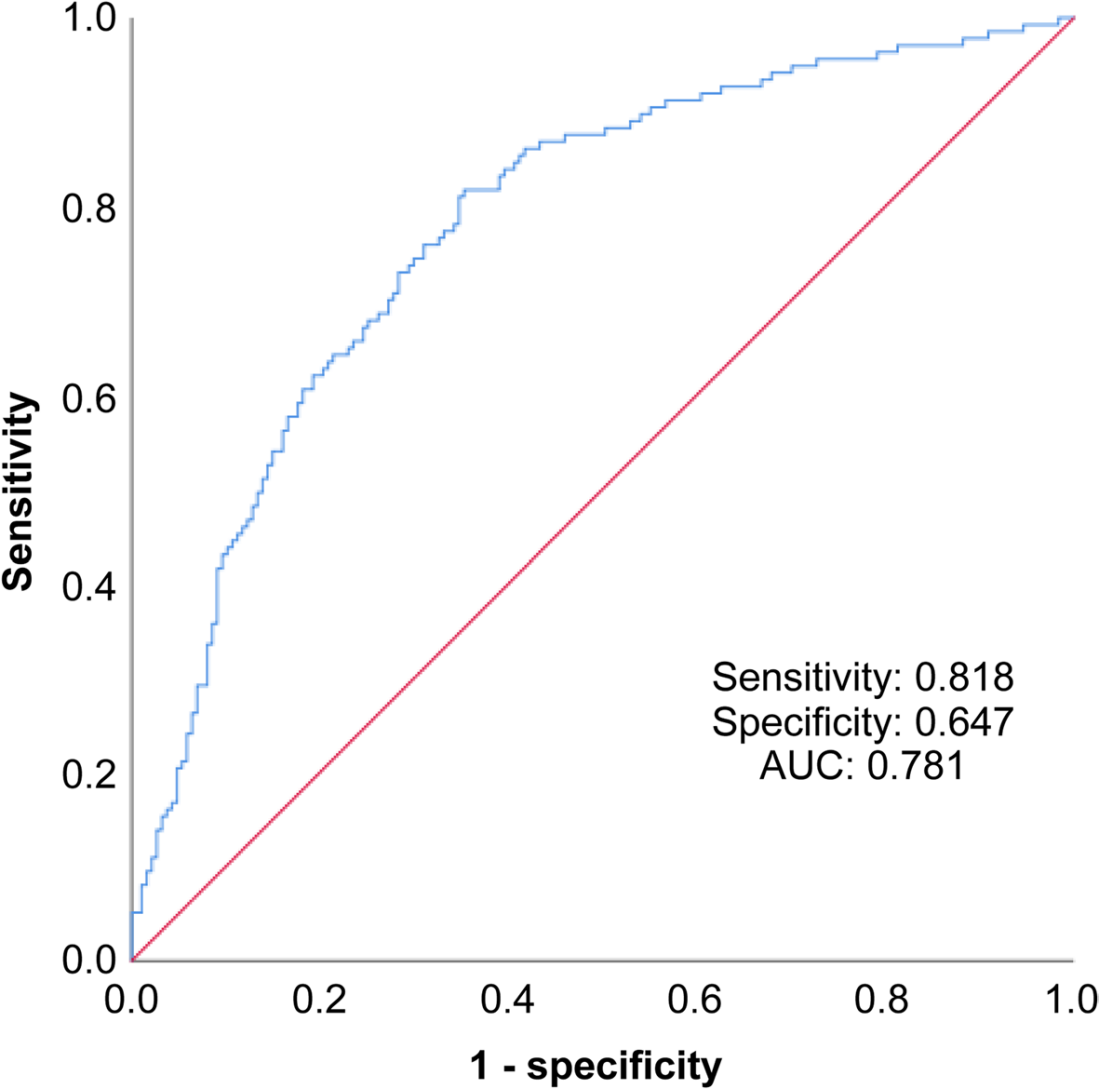
ROC curve built to determine the NLR cut-off value for the prediction of relapse in patients with NMOSD

**Fig. 3:**
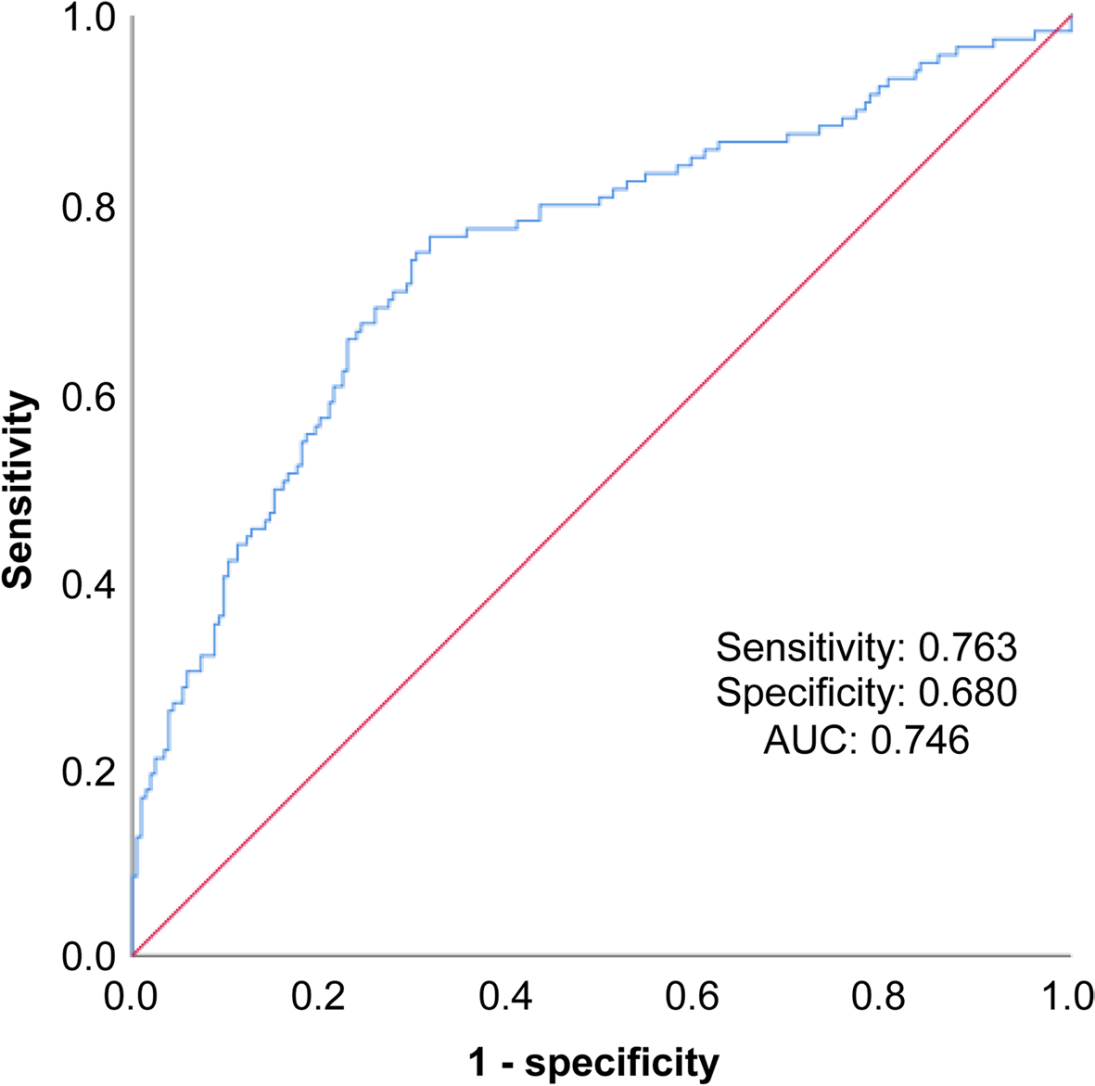
ROC curve built to determine the NLR cut-off value for the prediction of poor prognosis in patients with NMOSD

Based on the ROC curve, the optimal cut-off values of NLR for the prediction of the prognosis of NMOSD are as follows: relapse: optimal cut-off value of NLR = 2.38, sensitivity = 81.8%, specificity = 64.7%, area under the curve = 0.781, 95% CI = 0.731–0.832 (Fig. 2 for details); poor recovery: optimal cut-off value of NLR = 2.63, sensitivity = 76.3%, specificity = 68%, area under the curve = 0.746, 95% confidence interval = 0.688–0.804 (Fig. 3 for details). In addition, Table 5 shows the optimal cut-off values of various continuous variables for the prediction of relapse and poor prognosis in patients with NMOSD at the time of admission.

**Table 5:** Optimal cut-off values of various continuous variables for the prediction of relapse and poor prognosis in patients with NMOSD

	Relapse					Poor recovery				
	Cut-off point	Sensitivity	Specificity	AUC	P value	Cut-off point	Sensitivity	Specificity	AUC	P value
NLR	2.38	0.818	0.647	0.781	0.001	2.63	0.763	0.68	0.746	0.001
PLR	134	0.613	0.642	0.68	0.001	190	0.454	0.824	0.665	0.001
LMR	3.1	0.526	0.759	0.651	0.001	2.85	0.479	0.805	0.648	0.001
Initial EDSS	5	0.686	0.54	0.614	0.001	6	0.492	0.723	0.613	0.001

*EDSS* Extended disability status scale, *LMR* Lymphocyte-to-monocyte technical ratio, *NLR* Neutrophil-to-lymphocyte ratio, *PLR* Platelet-to-lymphocyte ratio

To further analyze the effect of clinical parameters on the relationship between NLR and prognosis, we performed subgroup and interaction analyses and found observed that NLR was positively associated with the prognosis for most variables. Furthermore, we found significant heterogeneity between subgroups analyzed according to sex, PLR levels and LMR levels, with a higher risk of relapse in female (OR = 1.43, 95% CI: 1.23–1.67, *P* < 0.001) than in male (OR = 1.14, 95% CI: 1.01–1.28, *P* = 0.034). Patients with low PLR levels (OR = 2.27,95% CI: 1.58–3.26, P < 0.001) had a greater risk of relapse with an increase in NLR levels than those with high PLR levels (OR = 1.15, 95% CI: 1.05–1.26, *P* = 0.004). We also found that Further, patients with high LMR levels (OR = 1.41, 95% CI: 1.18–1.70, *P* = 0.035) had a significantly higher chance of relapse due to elevated NLR levels than those with low LMR levels (OR = 1.11, 95% CI: 1.01–1.23, *P* < 0.001) (Tables 6 and 7).

**Table 6:** Effects of clinical parameters on the relationship between NLR and relapse

Variables	N	Relapse		
		OR (95% CI)	P value	P for interaction
Age, years				
< 60	261	1.25 (1.13–1.38)	< 0.001	0.107
≥60	63	1.64 (1.16–2.30)	0.0047	
Sex				
Male	93	1.14 (1.01–1.28)	0.0339	0.021
Female	231	1.43 (1.23–1.67)	< 0.001	
Hypertension				
No	279	1.28 (1.15–1.42)	< 0.001	0.851
Yes	45	1.24 (0.97–1.60)	0.0882	
Initial EDSS				
< 5	140	1.27 (1.10–1.46)	< 0.001	0.912
≥5	184	1.25 (1.10–1.42)	< 0.001	
Anti-AQP4 antibodies				
Negative	91	1.17 (1.03–1.33)	0.019	0.139
Positive	180	1.36 (1.17–1.58)	< 0.001	
LETM				
No	55	1.58 (1.11–2.23)	0.010	0.533
Yes	165	1.40 (1.20–1.64)	< 0.001	
Immunoglobulins				
No	291	1.29 (1.16–1.43)	< 0.001	0.908
Yes	33	1.26 (0.94–1.69)	0.120	
Immunosuppressants				
No	213	1.26(1.13–1.40)	< 0.001	0.516
Yes	111	1.36 (1.10–1.68)	0.004	
PLR				
< 134	173	2.27 (1.58–3.26)	< 0.001	< 0.001
≥134	151	1.15 (1.05–1.26)	0.004	
LMR				
< 3.1	117	1.11 (1.01–1.23)	0.035	0.017
≥3.1	207	1.41 (1.18–1.70)	< 0.001	
DMT				
NO	279	1.32 (1.18,1.48)		0.312
YES	45	1.18(1.00,1.39)		

Diabetes and hormones are too different between the groups, and the interaction is negligible

*EDSS* Extended disability status scale, *LMR* Lymphocyte-to-monocyte technical ratio, *NLR* Neutrophil-to-lymphocyte ratio, *PLR* Platelet-to-lymphocyte ratio, *LETM* longitudinally extensive transverse myelitis, *AQP4* aquaporin-4, *DMT* disease-modifying treatment started during the follow-up

**Table 7:** Effects of clinical parameters on the relationship between NLR and poor recovery

Variables	N	Poor Recovery		
		OR (95% CI)	P value	P for interaction
Age, years				
< 60	261	1.33 (1.19–1.49)	< 0.001	0.774
≥60	63	1.28 (1.10–1.63)	0.048	
Sex				
Male	93	1.26 (1.09–1.47)	0.003	0.520
Female	231	1.43 (1.23–1.67)	< 0.001	
Hypertension				
No	279	1.33 (1.19–1.49)	< 0.001	0.701
Yes	45	1.27 (1.02–1.57)	0.031	
Initial EDSS				
< 6	206	1.38 (1.19–1.61)	< 0.001	0.188
≥6	118	1.21 (1.07–1.37)	< 0.003	
Anti-AQP4 antibodies				
Negative	91	1.34 (1.10–1.63)	0.004	0.819
Positive	180	1.38 (1.19–1.60)	< 0.001	
LETM				
No	55	1.20 (0.96–1.50)	0.114	0.705
Yes	165	1.26 (1.12–1.42)	< 0.001	
Immunoglobulins				
No	291	1.31 (1.18–1.45)	< 0.001	0.573
Yes	33	1.46 (0.99–2.15)	0.057	
Immunosuppressants				
No	213	1.28(1.15–1.43)	< 0.001	0.274
Yes	111	1.46 (0.99–2.15)	< 0.001	
PLR				
< 190	234	1.44 (1.18–1.76)	< 0.001	0.090
≥190	90	1.18 (1.05–1.33)	< 0.001	
LMR				
< 2.85	96	1.28 (1.09–1.52)	0.003	0.728
≥2.85	228	1.24 (1.09–1.40)	< 0.001	
DMT				
NO	279	1.32 (1.18–1.48)	< 0.001	0.312
YES	45	1.18(1.00–1.39)	0.051	

Diabetes and hormones are too different between the groups, and the interaction is negligible

*EDSS* Extended disability status scale, *LMR* Lymphocyte-to-monocyte technical ratio, *NLR* Neutrophil-to-lymphocyte ratio, *PLR* Platelet-to-lymphocyte ratio, *LETM* longitudinally extensive transverse myelitis, *AQP4* aquaporin-4, *DMT* disease-modifying treatment started during the follow-up

**Fig. 4:**
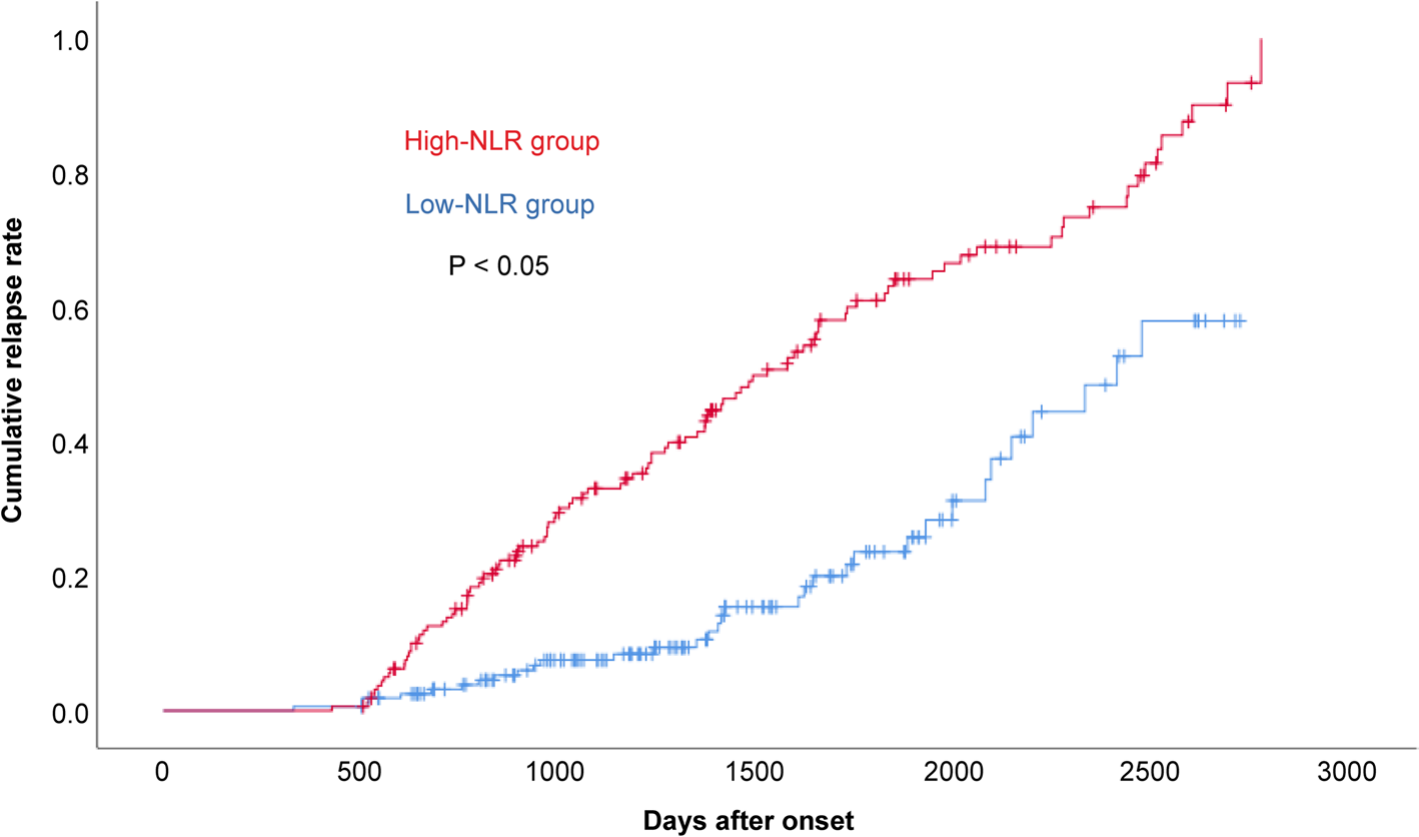
Cumulative relapse rate of high-NLR and low-NLR groups

Kaplan-Meier survival curve analysis and the correlation between cumulative relapse of NMOSD and NLR indicated that the relapse intervals of the high-NLR and low-NLR groups lasted 1525 days and 2409 days, respectively. The high-NLR group had a significantly lower median time to relapse than the low-NLR group (*P* < 0.001) (Fig. 4).

A Cox proportional hazards model was used to test the independent effect of NLR level on the relapse rate and poor recovery in NMOSD patients (Tables 8 and 9). A univariate analysis demonstrated that NLR level (HR = 1.05, 95% CI: 1.03–1.08, P < 0.001) was significantly correlated with the risk of relapse. Hypertension (HR = 1.70, 95% CI: 1.12–2.57, *P* = 0.013) and initial EDSS score (HR = 1.10, 95% CI: 1.008–1.20, *P* = 0.03) also affected relapse rate. After selecting variables, a multivariate Cox proportional hazards model revealed that NLR level (HR = 1.07, 95% CI 1.03–1.10, *P* = 0.001) remained a risk factor for relapse in patients with NMOSD. A univariate analysis revealed a significant relationship between poor recovery with NLR level (HR = 1.06, 95%CI: 1.04–1.08, P = 0.001). After correcting for confounding factors, multivariate analysis demonstrated a significant relationship between NLR level (HR = 1.08, 95% CI 1.04–1.11, P = 0.001) and poor recovery.

**Table 8:** Predictors of relapse in NMOSD patients by univariate and multivariable Cox proportional hazards models

Variable	Univariate		Multivariate	
	HR (95% CI)	P value	HR (95% CI)	P value
Age, years	1.00(0.99,1.01)	0.916		
Male sex, no. (%)	0.94(0.66,1.35)	0.738		
Hypertension, no. (%)	1.70(1.12,2.57)	0.013	1.31(0.80,2.16)	0.282
Diabetes, no. (%)	0.76(0.34,1.73)	0.518		
Initial EDSS	1.10(1.00,1.20)	0.03	1.05(0.94,1.16)	0.395
Anti-AQP4 antibody, no. (%)	1.43(0.95,2.13)	0.085	1.37(0.91,2.05)	0.127
LETM	0.89(0.55,1.45)	0.637		
Corticosteroids, no. (%)	0.78(0.34,1.76)	0.545		
Immunoglobulins, no. (%)	1.12(0.64,1.95)	0.686		
Immunosuppressants, no. (%)	1.34(0.94,1.91)	0.112		
NLR	1.05(1.03,1.08)	< 0.001	1.07(1.03,1.10)	0.001
PLR	1.00(0.99,1.02)	0.001	1.00(0.99,1.02)	0.881
LMR	1.01(0.98,1.04)	0.323		
DMT	0.72 (0.43,1.18)	0.19		

*EDSS* Extended disability status scale, *LMR* Lymphocyte-to-monocyte technical ratio, *NLR* Neutrophil-to-lymphocyte ratio, *PLR* Platelet-to-lymphocyte ratio, *LETM* longitudinally extensive transverse myelitis, *AQP4* aquaporin-4, *DMT* disease-modifying treatment started during the follow-up

**Table 9:** Predictors of poor recovery in NMOSD patients by univariate and multivariable Cox proportional hazards models

Variable	Univariate		Multivariate	
	HR (95% CI)	P value	HR (95% CI)	P value
Age, years	1.00(0.99,1.02)	0.47		
Male sex, no. (%)	0.96(0.65,1.42)	0.964		
Hypertension, no. (%)	1.28(0.78,2.10)	0.329		
Diabetes, no. (%)	1.40(0.71,2.77)	0.334		
Initial EDSS	1.12(1.01,1.23)	0.025	1.09(0.99,1.20)	0.094
Anti-AQP4 antibody, no. (%)	1.26(0.83,192)	0.280		
LETML	0.78(0.47,1.28)	0.318		
Corticosteroids, no. (%)	1.35(0.43,4.25)	0.609		
Immunoglobulins, no. (%)	1.03(0.55,1.92)	0.932		
Immunosuppressants, no. (%)	1.35(0.92,1.98)	0.127		
NLR	1.06(1.04,1.08)	0.001	1.08(1.04,1.11)	0.001
PLR	1.00(0.99,1.02)	0.001	1.00(0.99,1.02)	0.423
LMR	1.02(0.99,1.04)	0.218		
DMT	0.74 (0.44,1.25)	0.264		

*EDSS* Extended disability status scale, *LMR* Lymphocyte-to-monocyte technical ratio, *NLR* Neutrophil-to-lymphocyte ratio, *PLR* Platelet-to-lymphocyte ratio, *LETM* longitudinally extensive transverse myelitis, *AQP4* aquaporin-4, *DMT* disease-modifying treatment started during the follow-up

## Discussion

In the present cohort study, we collected large-scale clinical data on NMOSD and investigated its correlation with NLR. We found that NLR was significantly associated with relapse and poor prognosis in patients with NMOSD and hypothesized that NLR could be used as a biomarker to predict NMOSD prognosis.

The role of some neutrophils and lymphocytes in the pathogenesis of NMOSD has been found in previous studies. In the aquaporin 4-ab-positive NMOSD mouse model, neuroinflammation and loss of aquaporin 4 were reduced in neutropenic mice. In contrast, granulocyte colony stimulating factor increases the severity of NMOSD lesions. After using neutrophil protease inhibitors, damage is reduced [23]. In addition, previous research indicates that mice receiving AQP4 antibody-positive NMOSD patient IgG developed significant neuroinflammatory symptoms linked to neutrophil infiltration [24]. In patients with NMOSD, neutrophils are usually observed directly in the cerebrospinal fluid and CNS lesions [23, 25, 26]. These findings suggest that neutrophils play an important role in the pathogenesis of NMOSD. Numerous studies have demonstrated that B cells and T cells play important roles in the pathogenesis of NMOSD. Anti-AQP4 antibodies are usually produced by B cell subsets of CD19, CD27, and CD38 phenotypes [27]. CNS T cells activated by astrocytes also play an important role in disrupting the blood-brain barrier [28], and levels of Th17-related cytokines, including interleukin-21 (IL-21), IL-23, and IL-17, are significantly elevated in the sera of patients with NMOSD [29]; Th17-mediated responses and high Th17-related cytokine release have also been shown to be positively correlated with the degree of neurological disability in patients with NMOSD [30].

In another study, NLR was thought to predict all demographic, clinical, treatment-related, and psychosocial variables independent of an increase in MS-related disability [12]. The prognostic value of NLR in patients with NMOSD has not yet been deeply investigated, we hypothesized by statistical analysis that NLR could be used as a biomarker to predict NMOSD prognosis.

Our study included patients with first onset NMOSD, which allowed us to eliminate the effects of previous treatments. The mean age of NMOSD onset in our study was 43 years, which is consistent with that of a previous Spanish epidemiological study on NMOSD [20]. At the same time, we excluded children under 6 years of age because of their different blood cell count from adults, to avoid interference with the results. Anti-AQP4 antibodies can cause demyelination and neurological deficits by binding and damaging astrocytes [31], which have long been thought to be associated with prognosis as a unique biomarker of NMOSD. In the present study, we observed that the presence of anti-AQP4 antibodies was not associated with NMOSD prognosis; this finding is consistent with that of a recent study in that anti-AQP4 antibody titers do not reflect ongoing disease activity or subsequent neurological prognosis [32], a finding that may be related to the concentration of antibody titers. In addition, we detected a significant correlation between initial EDSS score and prognosis (*P* < 0.05), which illustrates the importance of early prevention and effective treatment. Finally, we classified the recovery of patients according to the EDSS score and observed that NLR values were significantly correlated with recovery. Patients with NMOSD with high NLR values had poorer recovery than those with low NLR values; findings also consistent with those of previous studies [33]. In another study, the time period to first relapse in patients with NMOSD lasted 14 months [34], and the mean follow-up interval was 43.9 months; therefore, our follow-up time was sufficient to permit reliable results. Previous well-known research indicates that the recurrence rate was lower among men than women. This is consistent with our stratified analysis results, which showed that women have a significantly higher risk of relapse than men [35]. Interestingly, we observed that as the NLR levels increased, the risk of relapse of low PLR levels was greater than that of high PLR levels, and the risk of relapse of high LMR levels is greater than that of low LMR levels. Unfortunately, we do not fully understand how this works. Therefore, our findings need to be validated in further studies with larger sample sizes.

We believe that widely accepted prognostic factors, such as LETM and AQP4 antibody were not significant in this study for two main reasons. First, our hospital did not conduct extensive AQP4 antibody detection before April 2016. Second, only a portion of the patients (67.9%) included in the study underwent spinal magnetic resonance examination, which is not widely representative, and may be related to the fact that LETM was not associated with a significant prognosis in this study. Furthermore, disease-modifying treatments (DMTs) were not an important predictor of prognosis. We considered that this may be due to the fact that a small number of patients chose to start DMT at the beginning of follow-up (13.9%). According to a large number of studies, early initiation of DMT is an important step to improve outcomes, and large prospective randomized trials in a larger population of patients are needed in the future.

The determination of NLR is easy, cost-effective, and less invasive, and its values are more stable than the values of leukocyte counts. PLR and LMR have also been mentioned in previous studies as biomarkers and have found applications in cancer and autoimmune diseases; therefore, we included PLR and LMR in the present study [36, 37]. In the comparative analysis of high-NLR and low-NLR ratios, we found a significant difference between PLR and LMR in both groups (*p* < 0.001); in the subgroup analysis we found that higher LMR levels and lower PLR levels caused a higher risk of poor prognosis with elevated NLR than did lower LMR levels and higher PLR levels. Therefore, in this study, although we could not obtain evidence suggesting that PLR and LMR can be used as biomarkers for the prognosis of patients with NMOSD, their role in determining the prognosis of patients with NMOSD cannot be completely excluded and further validation is needed.

Our study has several limitations because of its retrospective nature, considering that concomitant diseases and the treatment at the follow-up stage following the discharge of patients could not be thoroughly evaluated. Moreover, the set cutoff values to determine prognosis was affected by baseline EDSS. In addition, our data were derived from simple laboratory tests, and therefore, may be biased. Additionally, there are some conditions, not taken into account, that alter neutrophil counts, such as poor lifestyle habits of the patients. During our follow-up, 190 patients had lost contact, which may have incurred a selection bias in the study results. Furthermore, we did not conduct MOG antibody testing on all included patients, which may have affected the accuracy of the study results. Finally, anti-AQP4 antibodies were not measured in all included patients, which prevented a thorough assessment of the relationship between anti-AQP4 antibodies and prognosis. Therefore, more data, preferably from multicenter, long-term studies, are warranted before the use of NLR as a biomarker for NMOSD in clinical settings.

## Conclusions

Our study demonstrates that there is a significant correlation between NLR values and the prognosis of patients with NMOSD and that early monitoring of NLR values in patients with NMOSD is beneficial.

## Data Availability

The datasets used and/or analyzed during the current study are available from the corresponding author on reasonable request.

## Abbreviations

EAE: Experimental autoimmune encephalomyelitis
EDSS: Extended disability status scale
ELISA: Enzyme-linked immunosorbent assay
LMR: Lymphocyte-to-monocyte technical ratio
MS: Multiple sclerosis
NLR: Neutrophil-to-lymphocyte ratio
NMOSD: Neuromyelitis Optica Spectrum Disorder
PLR: Platelet-to-lymphocyte ratio
ROC: Receiver operating characteristic
AQP4: aquaporin-4
LETM: Longitudinally extensive transverse myelitis
DMT: disease-modifying treatment
